# Biomass-Based Composites for Agricultural Applications

**DOI:** 10.3390/polym17212851

**Published:** 2025-10-26

**Authors:** Yufeng Xie, Sen Ye, Yue Peng, Jiazhen Gao, Xiaoyun Li, Xingxiang Ji

**Affiliations:** 1 State Key Laboratory of Biobased Material and Green Papermaking, Qilu University of Technology, Shandong Academy of Sciences, Jinan 250353, China; 2 School of Light Industry and Engineering, South China University of Technology, 381 Wushan Road, Guangzhou 510640, China; 202421029256@mail.scut.edu.cn (Y.X.); 202220128201@mail.scut.edu.cn (J.G.); 3 School of Materials Science and Engineering, South China University of Technology, 381 Wushan Road, Guangzhou 510640, China; 202330380711@mail.scut.edu.cn (S.Y.); 202330380482@mail.scut.edu.cn (Y.P.)

**Keywords:** biomass, seed treatment, soil treatment, foliar treatment, controlled release

## Abstract

As chemical pollution and food safety risks in agriculture have increased due to global population growth and a food demand surge, the development of new environmentally friendly pesticide carriers is urgently needed to build a sustainable agricultural system. Owing to the excellent biocompatibility and controlled degradation of biomass materials and their specific interactions with active ingredients, biomass-based composites have unique advantages in the field of pesticide delivery. By regulating the carrier structure, the targeted controlled release of the pesticides can be achieved, leading to improvements in the chemical stability of the active substance and target absorption efficiency, and a significant reduction in environmental impact. This paper summarizes the innovative applications of biomass-based composites in agricultural scenarios, focusing on the breakthroughs in the three core areas of intelligent protection of seed coating, soil microcosm regulation, and foliar environment-responsive delivery. Through an in-depth analysis of the efficiency mechanism of composites on insecticides, antimicrobials, and herbicides, this review elucidates the scientific pathway of pesticide delivery through interfacial modification, slow-release kinetic modulation, and multilevel structural design, which will provide theoretical support and a practical paradigm for the development green agricultural technology.

## 1. Introduction

With the pressure on agricultural production triggered by global population growth and surging food demand, modern agriculture widely relies on chemical pesticides to enhance crop yields [1]. However, conventional pesticides are limited by their physicochemical properties, application techniques, and environmental factors, such as wind speed, temperature, and humidity, resulting in the loss of more than 50% of the active ingredients through evaporation, photolysis, etc. [2]. This not only aggravates soil and groundwater pollution and induces resistance in target organisms but also greatly reduces the utilization rate of pesticides [3]. Therefore, the research and development of new pesticide formulations with both efficient utilization and eco-friendliness has become a key breakthrough in the sustainable development of agriculture.

Seed, soil, and foliar treatments are commonly used pesticide treatments in modern agriculture, and are used in different stages of seed, soil, and plant growth to form a prevention and control system covering the whole life cycle of crops. Seed treatments guarantee healthy germination and early resistance through the triple mechanism of physical barrier construction, chemical–biological synergistic prevention and control, and biological stimulation [4]. Soil treatments and foliar treatments can be divided into three systems according to the different stimulus sources: endogenous stimuli [5] mainly include pH-controlled release [6], enzyme-responsive release [7], and so on; exogenous stimuli [8] mainly comprise temperature-controlled release [9]; multiple stimuli [10] mainly involves pH response and multiple response interactions such as enzyme response and temperature response. In addition, Soil conditioner in soil treatment agents can systematically optimize the three-phase structure of the soil to enhance soil fertility [11]. Foliar treatments can work by attaching to the leaf surface through adsorption [12]. This intelligent controlled release system promotes the evolution of pesticide treatment from crude applications to precise and intelligent directions [13].

The application of biomass-based materials in agriculture has attracted much attention because of their environmental friendliness and functional diversity. Their advantages are reflected in the following three aspects. Firstly, they are widely sourced and biodegradable, which significantly reduces environmental burden [14] and, as a pesticide carrier, they can regulate drug release, protect active ingredients, and promote absorption to achieve multifunctional integration, such as delaying or controlling drug release, stabilizing and protecting active ingredients, and promoting drug absorption [15]. However, they are limited by changes in physical and chemical properties, which may lead to a decrease in the pesticide release efficiency and stability [16]. For this reason, researchers have enhanced their performance through composite modification techniques; for example, polydopamine-modified biomass materials can enhance the foliar deposition of pesticides [17]. In addition, compositing biomass with other materials also improves the performance of soil amendments and promotes plant growth by increasing the water retention capacity and nutrient availability of the soil [18]. This type of composite system improves pesticide utilization and reduces environmental risks, thereby providing an innovative path for sustainable agricultural development.

**Figure 1:**
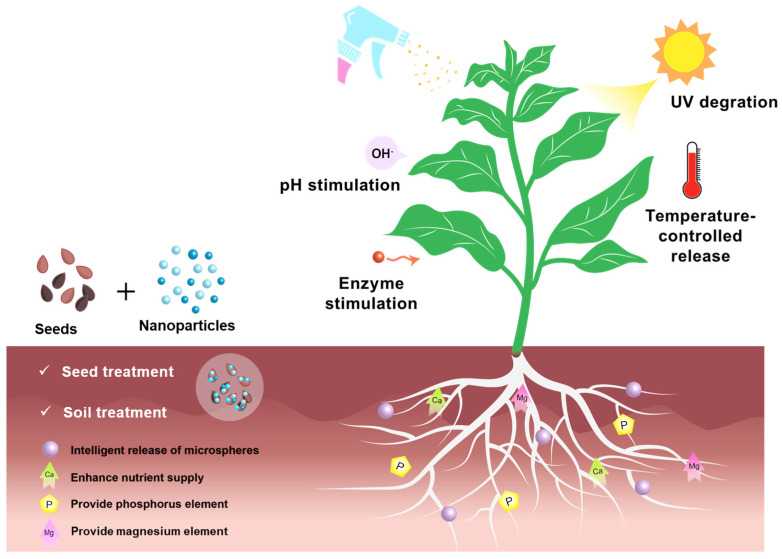
Application of biomass-based composite for agriculture.

In summary, seed, soil, and foliar treatments play an important role in modern agriculture, and the application of biomass materials provides new ideas for the sustainable development of agriculture (Figure 1). Compounding biomass with other materials can not only improve the performance of pesticides but also effectively reduce environmental pollution and promote the green development of agriculture [19]. Therefore, this paper reviews the effectiveness of a composite of biomass and other materials and an in-depth study is conducted regarding the mechanism of action of seed, soil, and foliar treatments, as well as their application in agriculture, which will provide more scientific and effective solutions for future agricultural production.

## 2. Seed Treatment Agent

Seed coating technology plays multiple roles in agricultural production by coating solid and liquid materials to form a functional layer on the seed surface [20]. Firstly, it regulates germination time and growth rate and promotes the synchronization of parental plant flowering to enhance hybrid seed yield [21]. Secondly, it serves as a carrier of nutrients, pesticides, and agricultural active ingredients; it has the composite functions of germination promotion, disease, and insect resistance [22,23]. Thirdly, increased seed weight can prevent wind dispersion and guarantee precise sowing [24]. Fourthly, through targeted delivery of active ingredients, the exposure of non-target areas can be reduced, which significantly improves the sustainability of drug applications and environmental protection [25]. This technology has been widely used in vegetable, field crop, and flower cultivation.

Biomass materials show unique advantages in the field of seed coating because of their excellent biocompatibility and degradability properties; special functional groups in their molecules can achieve the controlled release of active ingredients. Accinelli et al. [26] coated corn and rapeseed with starch-based bioplastics, with a composite of imidacloprid-metalaxyl-M insecticide and *T. harzianum spores* in the coating material. The germination of *T. harzianum-coated seeds* was significantly higher than that of the pesticide-containing control, and seedlings showed superior growth in terms of shoot height and root length. This growth-promoting effect was attributed to the efficient colonization of *T. harzianum* at the inter-root level, which was facilitated by the good compatibility and stable combination of the fungus and bioplastic coating layer.

Compared with dry powder coating technology, liquid film systems show significant advantages in seed treatment. On the one hand, its excellent film-forming properties can form a continuous and uniform protective layer to ensure full coverage of the seed surface [27]. It also possesses a high active ingredient loading capacity and precise slow-release kinetics and can be adapted to different seed forms to reduce mechanical damage [28]. Studies have shown that liquid films can effectively maintain seed water balance and provide an ideal microenvironment for germination. Larson et al. [29] used ethyl cellulose and gibberellic acid (GA) to construct the composite liquid coating to treat physiologically dormant seeds and found that the system extended the GA release cycle by 3.0- to 3.9-fold (compared to the control) and significantly enhanced *Penstemon palmeri* seeds at 15 °C and 25 °C, and had no significant inhibitory effect on seedling root length, shoot length, and biomass. By optimizing the synergistic effect of coating materials (e.g., ethyl cellulose) and plant growth regulators (e.g., GA), the attenuation of efficacy caused by the easy degradation/leaching of GA in traditional treatment methods is overcome, and an efficient solution is provided for ecological restoration projects by relieving the mechanism of seed dormancy.

**Figure 2:**
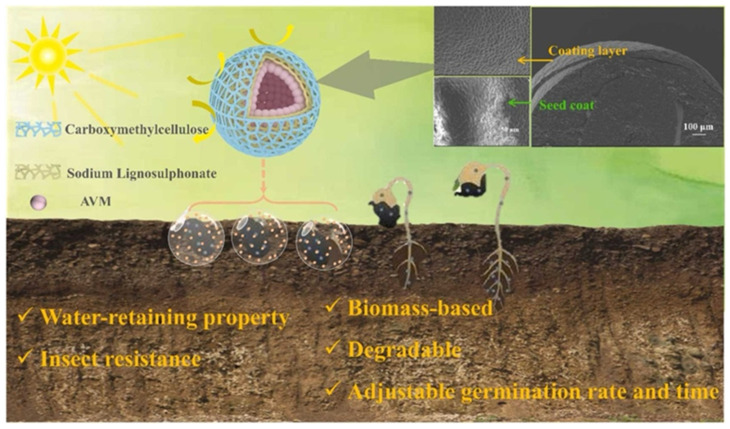
Application of carboxymethyl chitosan/sodium lignosulfonate composite membrane in seed coating (The yellow arrow indicates that it has excellent anti-ultravioleta ability.) [28].

Wang et al. [28] used carboxymethyl cellulose (CMC) as a film-forming matrix, enhanced its water resistance by formaldehyde cross-linking, and introduced sodium lignosulfonate (SL) as a functional additive (Figure 2). Under acidic conditions, formaldehyde and CMC hydroxyl groups undergo hydroxyl-aldehyde condensation. The abundant phenolic hydroxyl groups in SL are able to scavenge photooxidative radicals, and the presence of phenolic hydroxyl and sulfonic groups increases the strength of hydrogen bonds within the CMC molecular chain, thereby enhancing the mechanical strength of the film. Its benzene ring and conjugated structure endow the coating with excellent anti-ultraviolet ability. The coating thickness can be precisely controlled by regulating the soaking time, which not only improves the resistance of seeds to break through the film but also slows down the germination process by blocking the penetration of oxygen. The coating material was shown to be environmentally friendly, with a natural degradation rate of 80% within 60 days. Microstructural observations show that the coating layer covers the seed surface uniformly in a unique network shape and forms a water film to enhance the water absorption rate when exposed to water; at the same time, the AVM pesticide loaded in the film can be released through the micropores, which significantly improves the utilization rate.

In addition to the physical protection of seeds and controlled release of growth hormones, Xu et al. [30] prepared a biodegradable and adjustable biomass-based nanofiber from cellulose acetate for use as a seed coating to improve pesticide delivery and seedling development. The results of a greenhouse study showed that the nanofiber seed coating could deliver pesticides precisely to the target location with the use of a very small amount of pesticides. The Cu^2+^ releasing nanofiber coating promoted seed germination, especially under diseased medium conditions, and increased seedling biomass of the plants (12–29%). This approach can be used in pathogen-infested soil conditions to improve yield.

## 3. Soil Treatment Agent

Soil treatments play an important role as key inputs in modern agriculture for pest control, weed suppression, and soil microbiology regulation [31]. Current research focuses on the development of environmentally friendly treatments, in which innovative systems based on highly absorbent hydrogels have the potential to improve soil physicochemical properties in a multidimensional manner. This can improve soil growth conditions by enhancing soil water holding capacity, reducing irrigation, improving soil permeability, optimizing nutrient uptake, and slowing down the dissolution of fertilizers, thereby improving crop growth performance and yield [32].

Ahmad et al. [33] prepared an environmentally friendly cellulose-based hydrogel for controlled release fertilizer using cellulose raw material made from carboxymethyl cellulose and waste paper and epichlorohydrin as crosslinking agent. The cellulose hydrogel exhibited extraordinary water absorption properties (degree of swelling up to 20 times) and maintained 36.5%, 30.1%, and 23.4% of water content in topsoil, wet clay, and sandy soil, respectively, after seven days in soil moisture regulation tests. When used as a urea carrier, its slow-release efficacy was remarkable; the release of urea from different substrates was stable in the 71–75% range within 42 days, whereas more than 95% of conventional free urea was released within 7 days. These results suggest that urea-carrying cellulose hydrogels may be promising controlled-release fertilizers.

In addition to the common functions of water retention, swelling, and physical protection, the molecular-level environmental responsiveness of hydrogels provides new strategies for the development of agricultural materials that combine soil remediation with smart drug delivery [34]. For example, the β-1,4-glycosidic bond in cellulose can be hydrolyzed by cellulase to produce oligosaccharides or glucose. These oligosaccharides or glucans not only increase the soluble sugar content in the soil, but also promote the activity of soil microorganisms, thus improving soil fertility [35]. In addition, this degradation process can release drugs or nutrients encapsulated in the hydrogel, thereby enabling the diffusion and release of drugs [36]. Furthermore, through chemical modification, biomass macromolecules can be sensitive to slightly acidic or alkaline environments [37]. For example, modified lignin hydrogels undergo hydrolysis upon pH change to achieve responsive drug release, and this pH-responsive hydrogel has significant advantages in drug delivery systems to release drugs in specific acidic or alkaline environments and to improve drug utilization efficiency [38,39].

### 3.1. Endogenous Stimulation of Soil Treatment Agent

**Figure 3:**
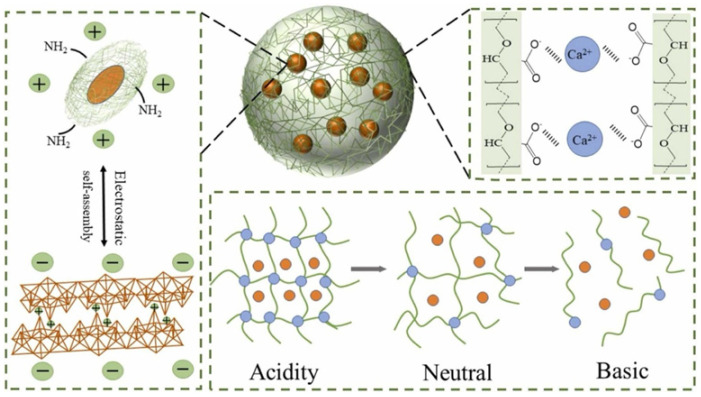
Polyethyleneimine-modified carboxy cellulose nanocrystals combined with acidified montmorillonite to achieve pH-controlled release of thiamethoxam [40].

pH is one of the most common endogenous stimuli; Dong et al. [40] constructed a pH-responsive thiamethoxam-loaded hydrogel (PEI-CCNC@A-MMT/TXM, Figure 3) by using an electrostatic self-assembly strategy of polyethyleneimine (PEI)-modified carboxy cellulose nanocrystals (CCNC) complexed with acidified montmorillonite clay (A-MMT) in combination with ionic cross-linking technique with sodium alginate. In this system, under acidic conditions (pH = 5), protonation of the carboxyl group (-COOH) formed a synergistic interaction between the primary hydrogen bond and the ligand bond, which resulted in densification of the gel network (the swelling ratios reached 10.0 times in 3 h) to achieve a slow and controlled release of the pesticide. In an alkaline environment (pH = 9), the carboxyl group dissociated into -COO^−^, and strong electrostatic repulsion triggered the complete disintegration of the gel within 90 min, triggering the sudden release of pesticides. Bacterial activity indicated that the hydrogel did not destroy the microbiological environment of the soil and had good biocompatibility.

Singh et al. [41] designed a millimeter-scale chitosan–alginate composite hydrogel (≈1.5 mm diameter) to achieve dual pH-responsive release of imidacloprid by introducing hollow microbeads to build a multistage pore structure. In acidic soil (pH = 5), chitosan amino acid protonation triggers electrostatic repulsion of the polymer chain segments, leading to an increase in pesticide release. In a neutral alkaline environment (pH 7.5), carboxylate ionization triggered a reconfiguration of the hydrogen bonding network, leading to an increase in pesticide release. This intelligent release mechanism based on the conformational transition of acid-base sensitive groups provides a novel delivery system design solution for adapting to soil environments with different pH gradients.

Compared to pH-responsive systems, which often take a long time to reach chemical equilibrium [42], enzyme-responsive mechanisms have significant advantages. Firstly, enzyme-catalyzed reactions, by virtue of their highly efficient enzyme-substrate-specific binding mechanism, can complete the response in milliseconds to seconds, and the rate of the reaction is controlled by enzyme activity and the concentration of the substrate [43], which is characterized by a rapid response. Secondly, the enzyme as a biocatalyst has molecular-level recognition accuracy for specific substrates [44], which can achieve precise regulation of biomarkers or metabolic pathways; thirdly, the enzyme molecule itself is naturally biocompatible and degradable [45], which is more suitable for the safety requirements of pesticide delivery scenarios. Pan et al. [46] successfully constructed abamectin by molecularly assembling 4,4-diphenylmethane diisocyanate with sodium carboxymethylcellulose, successfully constructing an avermectin (AVB1a) nanocapsule system. The average particle size of AVB1a nanocapsules was 352 nm, and the encapsulation rate was 92%. In terms of biological activity, the LC50 of the nanocapsules was as low as 0.82 mg/L against the *southern root-knot nematode*, which was 36% higher than that of the traditional emulsifiable oil formulation for the control of *root-knot nematode* diseases, and the cell membrane penetration of the target organisms and the horizontal/vertical soil mobility were significantly improved. Nanocapsules are 16 times less toxic to *earthworms* and cause less disturbance to the soil microbial community. This delivery system, which achieves precise release through enzyme response, combines the advantages of a simple preparation process, high targeting delivery efficiency, and controlled ecological risk and provides an innovative technological path for the development of green pesticides.

### 3.2. Exogenous Stimulation of Soil Treatment Agent

**Figure 4:**
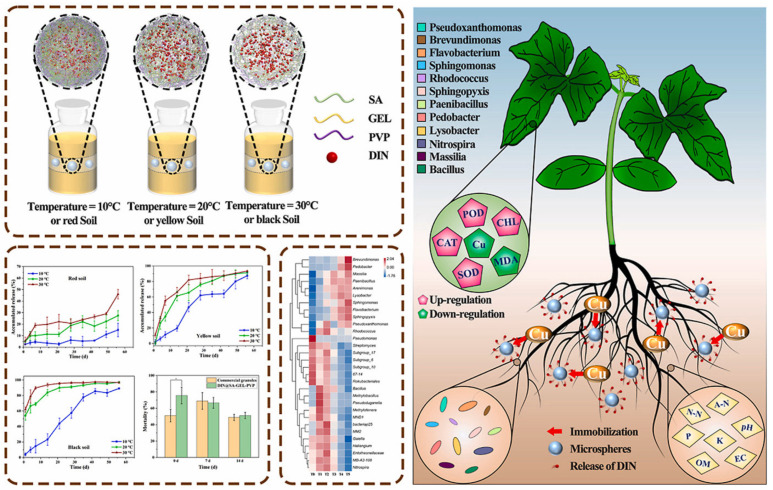
Sodium alginate-based composite microspheres for controlled release of pesticides and reduction in the adverse effects of copper in agricultural soils [50].

Endogenous thermoregulation relies on the plant’s own physiological metabolism and signaling mechanisms to achieve temperature control, which is usually slow to respond and takes a long time to produce significant effects [47]. Exogenous temperature control techniques can quickly respond to environmental temperature fluctuations and effectively maintain a stable temperature range for crop growth by means of artificial controls such as instantly turning on and off the cooling system or heating equipment [48,49]. Du et al. [50] investigated the preparation of furosemide (DIN) microspheres encapsulated with sodium alginate (SA), gelatin (GEL), and polyvinylpyrrolidone (PVP) composites and their performance, and the microspheres with an optimal drug loading of 19.77% were successfully obtained using a spray-drying technique (Figure 4). The microspheres demonstrated good stimuli-responsive controlled release properties under different temperatures and soil conditions and showed significant control effects against *Protaetia brevitarsis larvae* at the early stage of application, with the potential to inhibit pest outbreaks, especially at high temperatures. Blank microspheres can have a positive impact on the soil ecosystem, promoting the growth and physiological activity of cucumber seedlings, reducing the accumulation of Cu in leaves, enhancing soil nutrient levels, and effectively alleviating the problem of soil acidification. Further studies showed that blank microspheres could also increase the relative abundance of beneficial functional flora in the soil, enhance the ability of the soil to fix heavy metals, and promote plant growth. Redundancy and Spearman correlation analyses revealed that these beneficial functional bacteria were significantly positively correlated with soil conductivity, ammonium nitrogen, and nitrate nitrogen. The study concluded that the technology of combining physically modified carrier materials with pesticides is expected to reduce copper contamination in agricultural soils during pesticide application, which in turn reduces copper uptake by crops.

The application limitations of current controlled release fertilizers in the prevention and control of soil-borne diseases are as follows. Foliar application has absorption and conduction barriers, and irrigation can achieve precise delivery in the root zone, but it is accompanied by significant leaching and loss, which leads to environmental risks [51]. Root irrigation technology is difficult to apply to field crops such as soybean because of its high cost and complex operation; therefore, it is crucial to use controlled-release fertilizers more effectively and safely to control soil-borne diseases [52]. Optimizing the safe and efficient use of controlled-release fertilizers is especially critical, and agricultural ground film, which has the function of both weed control and disease prevention, as well as increasing efficiency and income, provides a new idea for this purpose [53]. Xie et al. [54] constructed a smart microcapsule (Pyr@MCC) by modifying microcrystalline cellulose to prepare a temperature-sensitive phase change material coated with pyrazole ether propyl ester (Pyr), and innovatively composited it with poly(vinyl alcohol)–starch film (PVA/ST), to develop a temperature-controlled release film (PM/PVA/ST). The system maintains a slow-release mode at 15 °C and triggers a rapid-release mechanism when the temperature rises to 35 °C, when Mycobacterium avium is active, to accurately match the needs of disease-outbreak environments. Both in vitro bacterial inhibition and potting tests verified the efficient control of *Fusarium*, confirming that this microcapsule-film composite system provides an environmentally responsive solution for controlling soybean root rot through an intelligent release mode.

### 3.3. Multi-Stimulation of Soil Treatment Agent

Compared with single-stimulus-responsive systems, multi-stimulus-responsive materials integrate the advantages of versatility, flexibility, and precision control through multidimensional synergistic regulatory mechanisms [55], which significantly enhances the efficacy of applications in complex environmental adaptations and scenario-specific needs. Bellemjid et al. [56] uses amidated low-methoxy pectin as a raw material constructed calcium pectinate/silica hybrid drug-carrying beads (CPG-Carb-SG, Φ ≈ 2.3 mm), whose innovation lies in the design of a time-controlled–environmentally responsive bimodal release system; the initial phase (first two weeks) achieves basal dose delivery through controlled erosion of the silica layer, and then switches to pH/temperature-responsive sol–gel-dominated release of the calcium pectinate matrix in the later phases—acidic soils with high temperatures (>25 °C) accelerated Fungicide release is accelerated by acidic soils and high temperatures (>25 °C), while neutral environments (pH 7–8) and low temperatures (<15 °C) slow release kinetics. This intelligent delivery system, which is precisely adapted to the recommended application cycle of carbendazim (10–15 days), reduces both the frequency of application and the risk of soil leaching, providing a technical solution to reduce groundwater contamination and phytotoxicity.

Huang et al. successfully prepared a UIO-66-NH_2_/SL bilayer pesticide carrier with a particle size of about 100 nm by chemically cross-linking sulfonic acid groups on sodium lignosulfonate (SL) with protonated UIO-66-NH_2_ [57]. The outer layer of this carrier loaded with thiamethoxam (TMX) can be used for early-stage pest control, while the SL and UIO-66-NH_2_ SL and UIO-66-NH_2_ formed a double protective layer to give excellent slow-release performance, while TMX in the micropores of UIO-66-NH_2_ in the inner layer was used for late-stage pest control. In addition, TMX could be rapidly released under alkaline conditions, presumably because alkaline conditions disrupted the chemical interactions between UIO-66-NH_2_ and SL, thus promoting TMX release. On this basis, the authors [58] further investigated the release mechanism of pesticides and found that higher temperatures could effectively promote the diffusion of pesticides from the carriers. Under acidic conditions, SL was protonated, resulting in weaker interactions between UIO-66-NH_2_ and SL, and the disruption of the outer SL structure enhanced the permeability of the composites, which significantly enhanced the diffusion release efficiency of thiamethoxam. Under alkaline conditions, the UIO-66-NH_2_ structure of the inner layer was destroyed and disintegrated in the alkaline solution, which also accelerated the release process of thiamethoxam. The results confirm that the double-layer pesticide carrier has good response characteristics to temperature and pH, which provides theoretical basis and technical support for its application as a controlled release pesticide carrier.

### 3.4. Soil Conditioner

Soil conditioner is a product that improves the physicochemical and biological properties of soil by adding specific substances, and its main function is to enhance the productivity and ecological health of the soil. It not only enhances soil fertility, but also optimizes soil structure and improves water retention and aeration, thus creating a more suitable growing environment for plants. Studies have shown that scientific soil improvement measures can significantly improve crop resilience and promote root development, thereby achieving double improvement in crop yield and quality [59].

Improvement of soil structure: Organic amendments (compost/humus, etc.) reconfigure the soil microstructure through cementation and agglomeration—their organic matter increment drives the formation of stabilizing agglomerates and builds a multistage pore network to optimize water and air transport [60], effectively preventing and controlling root rot and promoting root development. Isakovski et al. [61] showed that aqueous and biochar from beetroot filaments and manzanita significantly enhanced the delayed release and biodegradation process of organophosphorus pesticides (OPP) in sandy soils, an effect that was attributed to the key roles of aromatic structures and oxygen functional groups in stabilizing OPPs and their promotion of biodegradation. By immobilizing OPP-degrading bacterial strains on charcoal, the researchers observed a significant increase in microbial abundance and function within the soil column. Further 16S rRNA bacterial community analyses confirmed the important role of specific taxa in the biodegradation of organic compounds, including pollutants, and provided new ideas for the remediation of contaminated soils.

Enhancing nutrient availability: Inorganic amendments such as lime and phosphate fertilizers increase the availability of key nutrients by modifying soil chemistry [62]. Lime raises soil pH and reduces acidity, thereby increasing the solubility of calcium and magnesium [63]; phosphorus fertilizers provide phosphorus directly to plants, promoting root development and flowering and fruiting, which in turn improves crop yields and quality [64]. A study by Zhang et al. [65] demonstrated that combined organic–inorganic composting with chicken manure, maize stover, and oyster shell powder (OSP), with optimal additions of OSP at an optimum level of 40%, could effectively enhance the pH, organic matter, calcium, and magnesium content of acidic soil, thereby increasing crop yield. Corn stover, as a raw material for composting, provides a carbon source and promotes the growth and reproduction of microorganisms, which further enhances the effect of composting.

Promote microbial activity: Bio-amendment reconfigures soil ecological function through microbiome engineering strategy—microbial fertilizer enriches beneficial bacteria in a targeted manner, activates ‘decomposition–release symbiosis’ nutrient cycling engine, and promotes nutrient uptake by plants while driving the soil microcosm reconstruction. Soil microbial ecosystem restructuring [66,67]. To address the microbial inhibition caused by chlorpyrifos (CP) contamination [68], Yadav et al. [69] adopted a novel biofortification–detoxification coupling strategy. Biochar is produced from biomass waste produced after oil distillation. The incorporation of biochar increased the tolerance of *P. graveolens* in CP-contaminated soils by 28% (from 42 to 45% to 55–67%), and simultaneously reduced the accumulation of CP in plants and activated the activities of soil eco-enzymes, confirming that CP accumulation in soil was reduced by 17.6%, which was the most important factor in the development of soil ecosystems, confirming that the ‘vector–microbe–plant’ triad can break through the functional limitations of soil under pollution stress.

Improvement of water retention: Physical amendments regulate soil hydrodynamics through pore topology reconstruction engineering—sandy fractions optimize aeration and permeability to prevent staining, while bentonite builds a slow-release reservoir of dry-season water through interlayer hydration and swelling effects. Fabian et al. [70] prepared an acid whey–cellulose/polyvinyl alcohol (PVA) hydrogel from carboxymethyl cellulose sodium to improve the water retention capacity of soil. The addition of 5% PVA increased the biodegradation cycle of the gel by 60% and the application of 2% hydrogel in soil increased the water retention capacity by 19%. Field validation showed that the use of hydrogels in soil significantly favored the growth of the two plants tested (*white mustard* and *oilseed rape*). Seedlings grown in soil containing 1 PVA and 2.5 PVA hydrogels were 24.5 percent and 11.7 percent longer than control samples.

## 4. Foliar Treatment Agent

Foliar treatments, as a planting protection measure widely used in modern agricultural production, break through the restrictions of traditional soil application paths and achieve precise control of diseases and pests and optimization of crop physiological functions by targeting the regulation of microecology between leaves. The core of the technology lies in the construction of a environment-responsive release mechanism and biomimetic adhesion interface (nano carrier engineering/bio-interfacial modification), and this system can dynamically respond to the temperature, humidity, pathogen infestation, and other biotic stresses, and realize the high efficiency of the pesticide active ingredient enrichment and controlled-release transport on the leaf surface so as to maximally enhance the pesticide utilization rate and reduce the risk of being diffused into the environment.

Intelligent controlled-release systems sensitively regulate the release behavior of loaded pesticides by sensing changes in the surrounding environment (e.g., pH, temperature, enzymes, light and heat, acoustic waves, etc.) to produce specific responsiveness, leading to changes in the composition and conformation of their structures. Biomass has been widely used in drug carriers due to its excellent biocompatibility and easy biodegradation properties, which has the advantages of delaying or controlling drug release, stabilizing and protecting the active ingredients of the drug, promoting drug absorption, improving bioavailability, and aiding in drug targeting, etc. Schiavi et al. [71] isolated cellulosic constituents from hazelnut pruning shoots and shells by chemical bleaching and acid hydrolysis, and successfully synthesized cellulose nanocrystals; lignin nanoparticles were obtained by a solvent–anti-solvent method after fractionation of the lignin component. The experimental results showed that these two nanomaterials were not only able to inhibit the growth of *Xanthomonas xylosoxidans Xac* bacteria on leaves in vitro, but also reduced the morbidity rate with an effect comparable to that of copper oxide chloride.

### 4.1. Endogenous Stimulation of Foliar Treatment Agent

Endogenous stimulus-responsive nanomedicine carriers have become a research hotspot for precision agriculture drug delivery systems by virtue of their ability to specifically recognize pathological features of pest microenvironments (pH gradients, redox microenvironments, and enzyme activation, etc.).

#### 4.1.1. pH Stimulation Response

**Figure 5:**
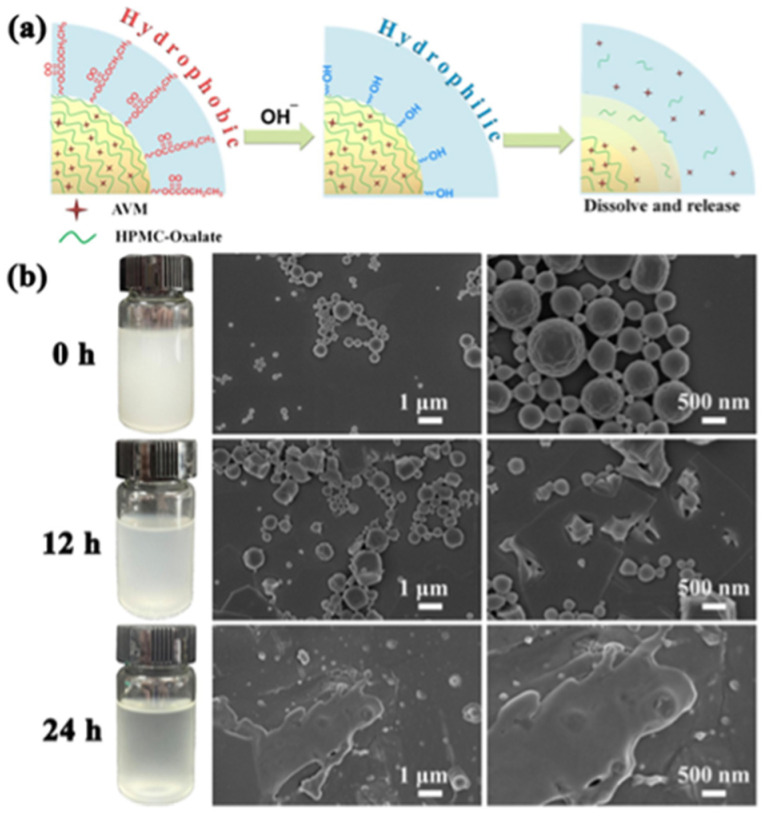
(**a**) Possible disintegration mechanism of AVM@HPMC-oxalate. (**b**) Macroscopic and microscopic morphological changes of AVM@HPMC-oxalate in pH = 9 buffer [72].

The pH-responsive smart pesticide delivery system achieves precise adaptation of pesticide release kinetics to the target microenvironment by constructing a pH gradient gating mechanism, e.g., the acidic microenvironment triggers the hydrophilic phase transition of carboxylate-functionalized carriers to drive the explosive release of pesticides, while the alkaline condition significantly slows down the release rate through the formation of hydrophobic barriers through the reorganization of intermolecular hydrogen bonding. Zhang et al. [72] used hypromellose to design an oxalate esterified hydroxypropylmethylcellulose (HPMC-Oxalate) delivery system; its neighboring carbonyl electron-deficient structure preferentially undergoes hydroxyl nucleophilic attack in the alkaline environment of the midgut of the pest, triggering a dynamic transition from a hydrophobic to a hydrophilic interface, which leads to the ‘exocytosis–introsolysis’ mode for the delivery of avermectin (AVM) to the focal site. The dynamic transition from hydrophobic to hydrophilic interface triggered the release of AVM in the mode of ‘external expansion and internal solubilization’ at the focal site (as shown in Figure 5).

The pH-responsive pesticide delivery system also constructs multiple regulatory mechanisms through molecular engineering strategies; on the one hand, the protonation breakage of acid-unstable chemical bonds (acetal/hydrazone bonds, etc.) is used to achieve targeted drug release in acidic environments. On the other hand, the charge-switching effect of ionizable groups (-COOH/-NH_2_) regulates the conformational change in the carriers. Ma et al. [73] employed an emulsion interfacial engineering strategy to build a composite material (AZOX@CMCS) coated with aminated silica nanoparticles (MSN) with high pyrimethanate (AZOX) loading rate and excellent fungicidal efficiency. The smart response mechanism of this system originates from the charge reversal effect regulated by the dynamic dissociation of carboxyl groups; at pH > 7, the increased dissociation of -COOH triggers strong electrostatic repulsion between -COO- groups, driving the polymer chain conformation from the curled state to the stretched state, forming a through-drug release channel. In addition, metal–ligand bonds can be used to make the materials pH-stimulated responsive in order to control pesticide release. Xu et al. [74] constructed a dopamine-Cu^2+^ chelating mesoporous system (AZOX@MSNsPDA-Cu) from dopamine hydrochloride to regulate the pesticide release, whose core mechanism lies in the dynamic coordination equilibrium between Cu^2+^ and azo bonds in the polydopamine layer. When pH < 6, H^+^ competitively coordinates with the dopamine catechol moiety, leading to the dissociation of the Cu^2+^–azo coordination network and triggering the explosive release of AZOX, whereas, under neutral conditions, Cu^2+^ forms stable hydroxyl groups with OH^−^ complexes under neutral conditions, which prolongs the release cycle of the drug and provides an innovative solution for the securing of heavy metals for agricultural use.

#### 4.1.2. Enzyme-Stimulated Response

Enzymes play an important role in living organisms, which are able to catalyze precise chemical reactions in metabolic processes under mild conditions. Therefore, the development of enzymes as triggers for the controlled release of pesticides has a number of advantages, including excellent specificity, selectivity, accuracy, and efficiency. Through enzyme-stimulated responses, pesticides are released only in the presence of specific enzymes, resulting in efficient and specific release while significantly reducing potential harm to non-target organisms and the environment. It is worth noting that when an external pathogen infests a plant, it secretes a variety of degrading enzymes (e.g., cellulases, hemicellulases, pectinases, proteases, and lignin-degrading enzymes), which are capable of breaking down components of the plant cell wall. Based on this property, the enzyme-stimulated response mechanism can be used to precisely control the release of pesticides, further improving the targeting and safety of pesticide use.

**Figure 6:**
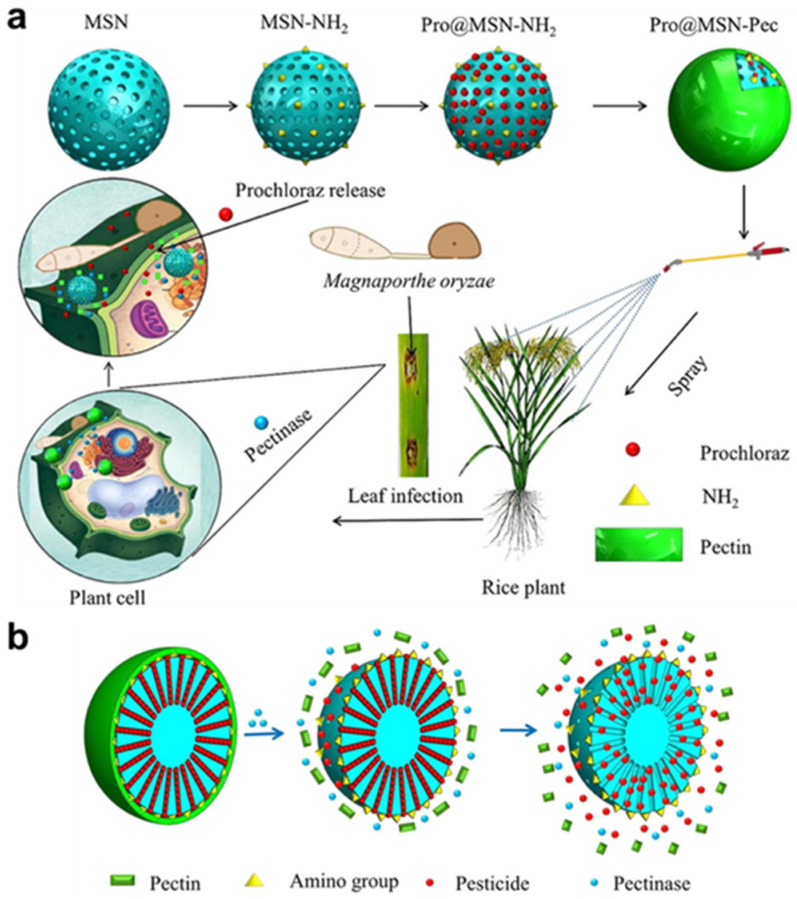
(**a**) Nanoparticles are used for pest control. (**b**) Schematic diagram of the release of imipramine from the nanoparticles [76].

Gao et al. [75] constructed a hydroxypropyl cellulose (HPC)-capped hollow mesoporous silica-based carrier (HMS-HPC), which utilized a synergistic triggering mechanism between the enzymatic hydrolysis of cellulose and the acidic microenvironment—cellulase specifically cuts off the β-1,4 glycosidic bond to dissociate the HPC layer, while the acidic condition accelerates the ester bond breakage to release the pesticides. Talat et al. [76], on the other hand, developed a pectinase-responsive smart coating system (prochloraz@MSN-pectin), whose pectin protective layer undergoes interfacial disassembly in? the presence of enzymes secreted by pathogens, triggering the opening of drug-carrying mesoporous channels (shown in Figure 6). Both types of systems improve pesticide utilization, reduce pesticide release in off-target areas, and provide programmable technological solutions for green plant protection by mimicking plant–pathogen interaction signaling pathways.

#### 4.1.3. REDOX Response

Redox-stimulated response is a mechanism for controlling pesticide release based on the differences in redox potentials in organisms, and typically involves materials containing disulfide bonds or other substances with multiple oxidation states (e.g., iron, selenium, and sulfur) as carrier materials. In organisms, these materials are able to fracture in the presence of reduced glutathione (GSH) to achieve redox responsiveness.

**Figure 7:**
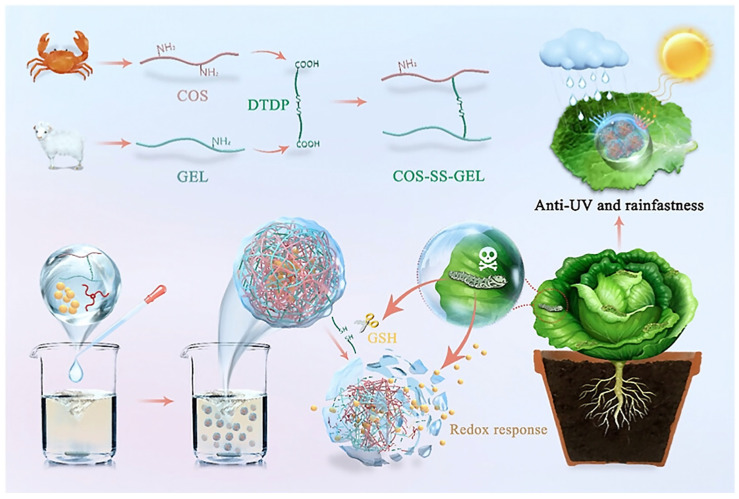
Redox-responsive nanopesticides [77].

Ma et al. [77] used 3,3′-dithiodipropionic acid (DTDP) crosslinked chitosan oligosaccharide (COS) with gelatin (GEL) to encapsulate the hydrophobic drug furosemide to construct nanoparticles. The core feature of the nanoparticle lies in function of the disulfide bond as a redox gating switch—the system maintains a stable and slow release (48.9% release rate at 100 h) when GSH is not added, and triggers thiol-mediated bond breaking when exposed to the GSH microenvironment, resulting in a jump of furosemide’s release rate to 97.8% at 100 h, and a 3.9-fold increase in release kinetic constants. Through the redox potential gradient recognition mechanism, this system can achieve the intelligent switching of ‘environmental silence-targeting explosion’, which provides a molecular-level regulatory paradigm for the development of smart pesticide formulations (Figure 7).

### 4.2. Exogenous Stimulation of Foliar Treatment Agent

Exogenous stimulus-responsive pesticide delivery systems achieve precise spatiotemporal control of release through a coupled multi-physical field regulation mechanism, the core of which lies in the design of smart materials with energy conversion properties (e.g., photo-thermal/magneto-thermal/acoustic-sensitive materials), which trigger a dynamic gating effect under the stimulation of exogenous sources such as a light field, a temperature gradient, an alternating magnetic field, or ultrasonic waves [78].

#### 4.2.1. Light Stimulation Response

Photoresponsive pesticide carriers use a light-controlled release mechanism; that is, in the dark or weak light conditions, the pesticide molecules are stably wrapped or adsorbed by the carrier. When the light intensity increases or a specific wavelength is irradiated, the carrier material undergoes a photochemical reaction, triggering structural changes and achieving targeted release of the pesticide molecules. This innovative mechanism is particularly suitable for agricultural scenarios that require light triggering, such as accurately releasing pesticides under conditions of sufficient daylight, which not only improves the utilization rate of the drug efficacy, but also effectively reduces the risk of potential pollution in non-target areas and the environment.

Gong et al. [79] grafted a photoresponsive coumarin derivative (CTC) onto a silica/PET composite membrane (SNM/PET) with an ultrathin ordered vertical nanochannel structure, and constructed a CTC@SNM/PET composite carrier based on light-controlled dynamic pore size regulation. The system uses alternating irradiation of 365/254 nm UV light to trigger a reversible cycloaddition/cleavage reaction of CTC to achieve intelligent opening and closing regulation of nano-channel aperture, thus precisely controlling the osmotic release behaviors of pesticide molecules. Yang et al. [80] developed a light-responsive intelligent delivery system based on β-cyclodextrin nanocapsules, encapsulating azobenzene derivatives and loading dimethoate to form a ternary composite system (β-cyclodextrin/azobenzene/dimethoate) which can achieve light-controlled drug release. Under UV or visible light excitation, the azobenzene molecule undergoes a trans–cis conformational transition, which destroys the original host–guest interaction, leading to the disintegration of the nanovesicle structure and the precise release of the pesticide active ingredient. This light-triggered conformational transition controlled-release strategy enables environmentally responsive pesticides to be delivered on demand and significantly improves the spatial and temporal targeting of the active ingredients.

#### 4.2.2. Temperature Stimulation Response

Temperature-responsive pesticide carriers belong to the exogenous stimulus-responsive intelligent drug delivery system, whose core mechanism lies in the thermotropic dynamic phase change behavior of the carrier material; through the temperature-induced hydrophobic-hydrophilic equilibrium change, the carrier can dynamically regulate the structural state of the density. When the ambient temperature is lower than the critical phase transition point, the polymer chain shrinks to form a dense barrier to inhibit the diffusion of pesticides. When the temperature rises to the threshold, the material undergoes conformational stretching and improves the porosity, which accelerates the release of pesticide molecules. This temperature-sensitive property is coupled with the occurrence of plant diseases and pests and the change in environmental temperature, so that the pesticide can automatically enhance the release intensity during the active period of high-temperature diseases, thus constructing a positive feedback controlled-release mode of ‘increasing temperature–accelerating the release–enhancing the efficacy of the pesticide’, and providing a new way to establish an environmentally matched precision application technology.

**Figure 8:**
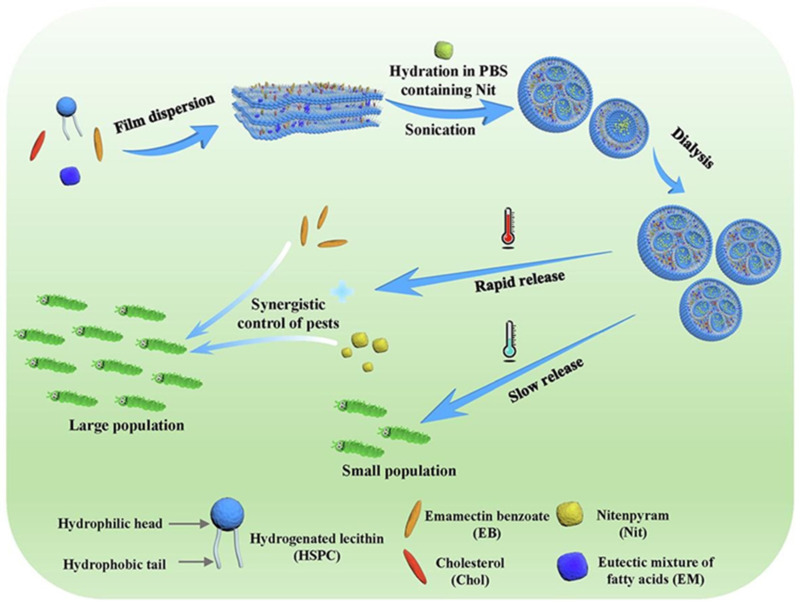
Preparation of Co-EM-Lip and its use for temperature-sensitive controlled release [81].

Du et al. developed a temperature-sensitive dual-pesticide co-delivery system based on fatty acid phase-change modulation; the synergistic loading of the water-soluble pesticide methamidophos benzoate and the fat-soluble pesticide allylcarbamate was successfully achieved through the construction of a multicompartmental liposomal nano-vesicle modified with a low eutectic mixture of decanoic acid-stearic acid (EM) (Co-EM-Lip) [81]. The system uses the phase transition threshold of 22 °C for EM materials to construct an intelligent controlled release mechanism; when the ambient temperature is lower than the threshold, solid EM stabilizes the bilayer structure of liposomes and when the temperature rises above 22 °C, EM melting triggers disorders in the arrangement of phospholipid molecules on the surface, which accelerates the simultaneous release of the dual pesticides through the dynamic reconfiguration of the membrane structure. The experiment confirmed that the insecticidal efficiency under the high-temperature environment of 35 °C was 4.4 times higher than that of 15 °C, showing the intelligent response characteristics of temperature–release efficacy, which provided an important solution for the development of environmentally adaptive precise-controlled pesticide release technologies (shown in Figure 8).

#### 4.2.3. Magnetic Field Stimulation Response

Magnetic field-stimulated pesticide release technology is an innovative method to precisely regulate pesticide release through an external magnetic field. The technology uses magnetic materials such as superparamagnetic iron oxide nanoparticles, which are embedded in a polymer, lipid, or protein carrier to form a magnetic composite carrier. When an external magnetic field is applied, the carrier can trigger the release of pesticides through magnetogenic heating or structural deformation, and its release position and dose can be controlled in the strength/direction of the magnetic field. For example, the local magnetic field can be directed to aggregate nanoparticles and release pesticides to achieve precise targeted treatment of pests and diseases. At the same time, the magnetic carriers can be recycled through the magnetic field to significantly reduce the environmental residual pollution.

**Figure 9:**
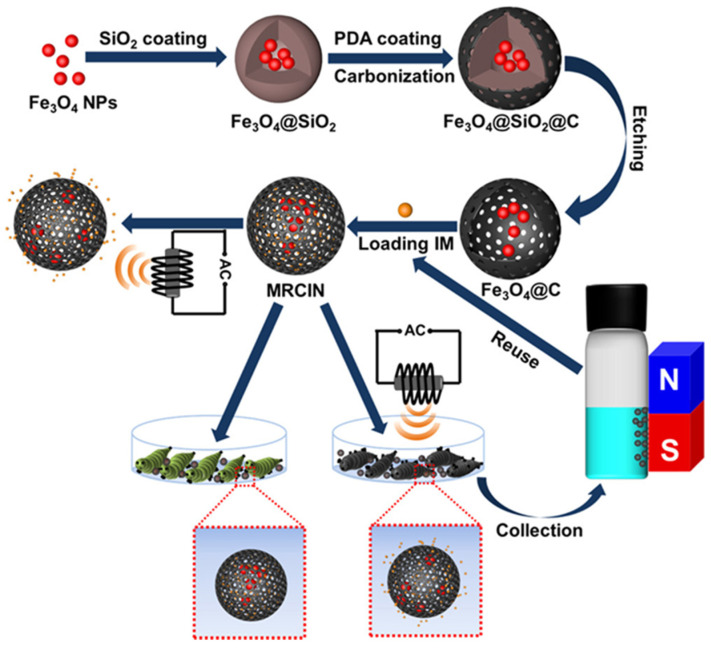
Schematic representation of the build and release performance of MRCIN [82].

Zhang et al. [82] designed an imidacloprid nanocarrier (MRCIN, Figure 9) based on the yolk shell structure Fe_3_O_4_@C. By combining the magnetic Fe_3_O_4_ core with the amorphous carbon shell, an intelligent delivery system with controlled-release characteristics in response to an alternating magnetic field was constructed. In this system, the Fe_3_O_4_ core can generate mechanical vibration under the action of electromagnetic field to effectively regulate the release kinetics of imidacloprid, which is significantly different from the burst release mode of traditional formulations. Meanwhile, the outer carbon shell not only serves as a drug-loading matrix, but also protects the Fe_3_O_4_ core from environmental corrosion and oxidative degradation by acting as a physical barrier, so as to maintain the long-lasting stability of the system.

### 4.3. Multi-Stimulation of Foliar Treatment Agent

With the advancement of single-stimulus-responsive carrier technology and in-depth analysis of crop pest and disease mechanisms, researchers have developed more complex intelligent pesticide controlled-release systems. These multi-stimulus response carriers can achieve precise activation and dynamic regulation of pesticide release by integrating various environmentally sensitive components such as pH, light, enzyme, temperature, and redox potential. Its innovative design not only gives it the dual functions of target recognition and precise release, but also significantly improves the selectivity and timeliness of pest control through the synergistic effect of multiple stimuli and promotes the development of pesticide delivery system into an environmentally adaptive intelligent platform.

The multi-responsive pesticide delivery system based on endogenous stimulation has significant application advantages. It realizes a precise targeted activation and release mechanism by autonomously identifying biomarkers specific to the pest microenvironment (such as local pH gradient, GSH, or pathogen-specific enzymes). On the other hand, compared with the controlled release technology, which relies on exogenous stimulation such as light and magnetic field, the endogenous response mode does not require the intervention of external equipment and is more suitable for complex farmland scenes. Zhao et al. [83] developed an environmentally friendly smart pesticide carrier, CS-SS-Zein, based on natural polymers by bridging chito-oligosaccharides and zein through a dynamic covalent bond of 3,3′-dithiodipropionic acid (DTDP). The vector efficiently loads avermectin through hydrophobic interaction with hydrogen bonding and enables rapid drug release at pH/GSH trigger. On the other hand, Yu et al. [84] designed a composite carrier with adjustable wall thickness by alternating the deposition of chitosan and sodium lignosulfonate at the interface of alkaline lignin-based Pickering emulsions. The system not only significantly improves the physical stability of the emulsion, but also precisely regulates the release of avermectin through the dual stimulation response of pH/laccase, which can enhance the efficiency of targeted delivery of pesticides and reduce environmental risks.

The core advantage of the multi-response pesticide carrier constructed by combining endogenous and exogenous stimuli lies in the organic integration of environmental intelligent sensing and artificial precision intervention through multimodal response logic; endogenous stimuli (e.g., pathogen-specific enzymes, local pH anomalies) endow the system with target activation capability under natural conditions, ensuring that the pesticide is triggered to be released autonomously in the microenvironment of the pests and pathogens; while exogenous stimuli (e.g., temperature gradient, magnetic field or light) serve as compensatory regulators to effectively overcome the limitations of fluctuating endogenous signal strength (e.g., soil pH heterogeneity) or insufficient response thresholds in the field environment. This synergistic controlled release paradigm breaks through the limitation of the linear response of the traditional single-stimulus system, and provides an innovative solution for the construction of an intelligent plant protection system that combines precision, robustness, and sustainability.

**Figure 10:**
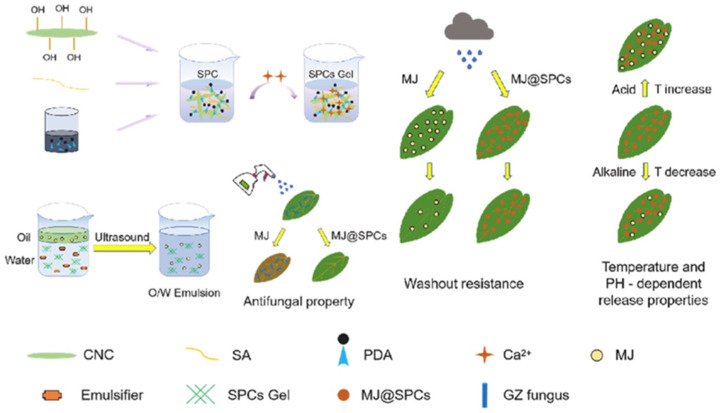
Schematic representation of MJ@SPCs preparation [85].

Ma et al. [85] developed a biocompatible SPCs gel with an interpenetrating network structure through the multicomponent synergistic assembly of sodium alginate (SA), polydopamine (PDA), cellulose nanocrystals (CNC), and loaded with pyrimethanil (MJ) to form a stable nanoemulsion, MJ@SPCs. The system exhibits a unique pH/temperature dual-responsive property; at low temperatures, the dynamic hydrogen bonding network between hydroxyl groups and imines in the gel network captures a large number of water molecules, causing the system to exhibit a hydrophilic and swelling state, and delaying the release of MJ through the physical barrier effect; when the temperature increases, the thermal perturbation disrupts the hydrogen bonding equilibrium, which prompts the exposure of the intramolecular hydrophobic groups and their remodeling, resulting in the formation of a dense hydrophobic layer, leading to the contraction of the gel size and the opening of the drug diffusion channel, which significantly accelerates the release of MJ (Figure 10).

### 4.4. Foliar Adhesion

Efficient deposition and stable retention of pesticides on crop foliage is a key challenge to improve pesticide utilization and reduce environmental risks. The inherent superhydrophobicity of crop foliage is due to the double barrier on the surface; a low surface free energy waxy layer at the chemical level significantly reduces droplet wettability, and the composite micro/nanoscale topology (e.g., papillae or grooves) at the physical level further strengthens the apparent contact angle through the Cassie–Baxter effect, resulting in the typical ‘Lotus Leaf Effect’. This synergistic defense mechanism makes it difficult for conventional pesticide formulations to spread effectively, and most of the liquid is lost during spraying due to bouncing, aggregation, or runoff, especially during the rainy season when the losses are more serious.

To address this core problem, modern pesticide formulation engineering focuses on the ‘target surface adaptation’ strategy, which breaks through the superhydrophobic barrier on crop foliage through chemical–physical bimodal synergistic mechanisms. Its core pathways can be divided into two categories: chemical bonding to enhance interfacial affinity and topological interlocking to strengthen mechanical anchoring. For the chemical bonding, based on the principle of bionic adhesion, designing carriers containing polyphenol hydroxyl groups (e.g., dopamine), catechol (e.g., tannic acid), or cationic groups, and through hydrogen bonding, π-π stacking, and charge interactions, directional non-covalent bonding occurs with the alkane/ester components of the foliar waxy layer, so as to reduce the pesticide contact angle to less than 30°, and significantly enhances the initial deposition efficiency. Topological interlocking refers to the use of the size-matching effect of the papilla/groove microstructure unique to the leaf surfaces of crops such as rice and wheat to construct a fibrous porous network structure or concave carrier to form a “mortise and tenon”-type physical interlocking with the leaf surface. For example, silica-based microspheres modeled on lotus leaf papillae can be embedded in the microstructural gaps of the foliage, resulting in a significant reduction in the rate of rainfall loss through erosion.

#### 4.4.1. Chemical Bonding to Enhance Interfacial Affinity

Through the biomimetic interface design strategy, hydroxyl, carboxyl, and other foliar affinity functional groups are introduced onto the surface of the pesticide carrier, which can form a strong adhesion interface with the high-level fatty alcohols and acids in the waxy layer of the plant foliage through intermolecular interactions such as hydrogen bonding and van der Waals force, so as to reduce the contact angle of the drug carrier system with the foliar surface to less than 30°, and significantly enhance the wetting spreading performance. Through precise chemical modification, this kind of functionalized drug-carrying system not only realizes the directional anchoring of pesticide active ingredients on the leaf surface and improves the ability of resistance to rainfall, but also improves the pesticide utilization and dosage by regulating the stability of the dispersion.

Through the design of bionic adhesion mechanism, polydopamine can effectively enhance the foliar deposition of pesticides due to its catechol moiety that can form multiple intermolecular hydrogen bonds with foliar high-level fatty alcohols/acids. He et al. [86] developed polydopamine-modified mesoporous nanoballs (PDAMNBs) and loaded them with pyrimethanil in situ through solvent-mediated polymerization-induced self-assembly. The carrier significantly enhanced foliar wettability by reducing the dynamic contact angle of the liquid in tomato leaves from about 65° to 24° through surface topology optimization. Its micro- and nanocomposite structure further led to a reduction in liquid splash loss.

In addition to dopamine, natural compounds with polyphenolic hydroxyl structures are becoming the preferred materials for pesticide functionalization carriers, among which tannic acid (TA) shows multidimensional potentiation potential due to its unique chemical properties. As a plant-derived polyphenol, TA not only achieves dopamine-like oxidative self-polymerization through o-phenyltriphenol groups, but also forms a highly stable coordination network with metal ions, such as Fe^3+^, which imparts antimicrobial activity and interfacial adhesion to the system at the same time. Based on this property, Chen et al. [87] constructed a tannic acid–iron ion composite functionalized titanium dioxide column-supported montmorillonite drug-carrying system, in which TA phenolic hydroxyl groups formed dynamic hydrogen bonding with the foliar waxy layer, increasing foliar deposition from 2.90 mg/cm^2^ in AVM to 15.50 mg/cm^2^, a 4.34-fold enhancement.

In the pesticide carrier engineering of natural polyhydroxy compounds, polysaccharides and protein-based biomolecules are becoming emerging materials for the construction of target-affinity delivery systems by virtue of their unique molecular topology and functional group distribution. Polysaccharides, represented by cellulose and starch, can be anchored by multi-point hydrogen bonding with ester and hydroxyl groups in the leaf surface wax layer through the high-density hydroxyl network formed by β-1,4 glycosidic bonding or α-1,4/1,6 glycosidic bonding [88], whereas plant proteins, such as zeinolysin, make use of polar amino acid residues (e.g., amide groups in glutamine) in their amphiphilic helical structures to produce a van der Waals force synergistic effect with the hydrophobic regions of the leaf surface [89]. These natural carriers not only achieve differential recognition of surface chemistry with non-target organisms (e.g., bees), but their biodegradability also significantly reduces the risk of microplastic accumulation in the soil, providing a green solution for sustainable agriculture.

#### 4.4.2. Topology-Based Adhesion

The microstructure of crop leaf surfaces is jointly regulated by the surface wax layer and the degree of epidermal cell wall development, forming amorphous, reticulate, or multilevel three-dimensional structures. When the wax density of the leaf surface was low, it was easy to form a smooth amorphous wax layer; with the increase in wax density, the wax layer gradually transformed to a fine three-dimensional structure. The foliar microstructure was mainly characterized by papillae, grooves, and cracks, which significantly affected the droplet spreading behavior by increasing the surface roughness. It is worth noting that leaves with multi-level structures (such as nano-scale protrusions superimposed on micron-scale substrates) can significantly enhance the physical adhesion of particles on the leaf surface; this has important application value for the efficient attachment and long-term retention of pesticides on the leaf surface.

Straw ash-based biocarbon silica composites (BCS) exhibit excellent adsorption capacity and stability by virtue of the micro/nano porous structure, high specific surface area, and negative surface charge, and at the same time, have the function of promoting plant growth. Cai’s team [90] used straw ash as a raw material to prepare BCS, and introduced BCS with a particle size of 10 μm into the pesticide system, and constructed a functional pesticide formula with a special microstructure, which could be used as a reference for the development of agricultural chemicals; these formulations are loss- and rain-wash-resistant. It was found that the leaf surface of the target plant, witch hazel, has a unique dual micro-nanostructure, with macroscopically distributed star-shaped villi and microscopically disordered waxy nano-crystals, and the BCS formed a mechanical interlocking effect with the leaf surface through the porous and rough surfaces—the nanorod gaps create a physical barrier, and the fine pores enhance the adsorption through capillary action, thereby achieving a significant increase in adhesion performance on hydrophobic foliage.

In addition, functionalized pesticide carriers can be prepared to match and complement the unique micro- and nanostructures of target surfaces to improve the retention and deposition of pesticides on crop foliage. Zhao et al. [91] developed a cap-shaped Janus carrier (HJC) using emulsion interfacial polymerization, which has a unique concave geometry and a unique porous surface (diameter 2.2~2.6 μm), and constructed a multistage rough structure consisting of 40–200 nm nanospheres on the surface. When applied to rice foliage, HJC can be effectively embedded into the microscopic nano-topography of the leaf by virtue of its cap-like topological properties, demonstrating excellent topological aptitude. Experimental data showed that the maximum retention of drug-carrying HJC on the leaf surface could reach 30.31 mg/cm^2^ (Wilhelmy method) and 12.51 mg/cm^2^ (40° spray), which fully proved its good retention performance and scouring resistance.

#### 4.4.3. Synergistic Adhesion by Molecular Recognition + Mechanical Adaptation

**Figure 11:**
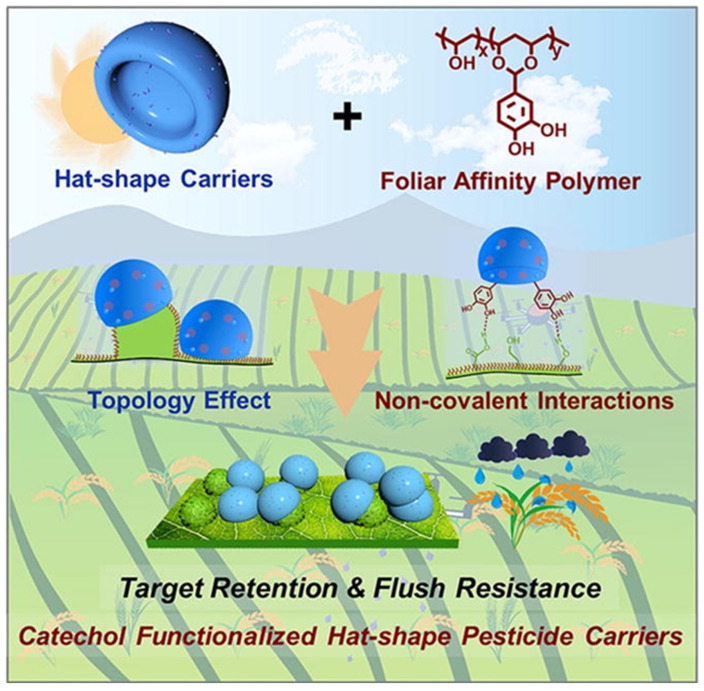
Catechol-functionalized cap carriers for prolonged pesticide retention time and scouring resistance on leaves [92].

It is worth noting that the above-mentioned two strategies can play a synergistic role through the design of composite carriers. The pH-responsive foliar iron fertilizer developed by Wang’s group [92] utilizes the dual function of carboxylated microcrystalline cellulose; the surface carboxyl group is complexed with Fe^2+^ and the hydroxyl group forms a hydrogen bond coating layer with attapulgite. In the acidic environment, the complex iron ions are dissociated synchronously and the mineral layer is loosened to realize the controlled release. Thanks to the spatial barrier effect of the clay, the fertilizer deposits on the foliage of maize were significantly higher than those of conventional formulations, and the resistance to rainwater washout was enhanced. Based on a similar design concept, the researchers used a surface modification strategy to graft P(VA-g-HBA) polymers containing catechol groups, combining non-covalent synergistic effects with the ‘hanging cap’ topology effect to precisely target the functionalized carriers on the surface of rice/wheat leaves (Figure 11). Experiments showed that the affinity of the system for foliar micro-nano-fragments was significantly enhanced, and the pesticide retention and scouring resistance were increased by about 30%, which verified the universal advantages of the composite carrier design in agricultural applications.

## 5. Conclusions

This paper has reviewed the application of biomass composites in agriculture, especially the innovative practices in seed smart coating, soil ecological regulation, and foliar precision delivery. Biomass composites can improve the release efficiency of pesticides, such as intelligently controlling the release of pesticides through stimuli such as pH or enzyme response, thereby improving the utilization of drugs, reducing drug residues in the environment while increasing economic efficiency. It can also improve the yield and quality of crops by promoting the absorption of nutrients by plants and enhancing the stress resistance of crops. In addition, the diversity of biomass materials enables them to achieve multiple functions in pesticide carriers, which promotes the green development of agriculture. With the increasing demand for sustainable agricultural development, the research and application of biomass composites will provide more scientific and effective solutions for future agricultural production. Through continuous exploration and innovation, biomass composites will play an important role in achieving sustainable agricultural development, protecting the ecological environment and ensuring food safety. In the future, biomass composites are expected to further optimize their intelligent response mechanism and functional synergy through multidisciplinary integration and the integrated innovation of cutting-edge technologies and realize large-scale application in the whole agricultural industry chain. At the same time, we should overcome the technical bottlenecks such as cost control and long-term stability, and build a solid material foundation for the construction of an environmentally friendly, resource-saving, efficient, and safe modern agricultural system, leading the sustainable development of agriculture to a new frontier.

## Data Availability

No new data were created or analyzed in this study.
